# Automated Android Malware Detection Using User Feedback

**DOI:** 10.3390/s22176561

**Published:** 2022-08-31

**Authors:** João Duque, Goncalo Mendes, Luís Nunes, Ana de Almeida, Carlos Serrão

**Affiliations:** 1 ISTAR-Iscte, Iscte—Instituto Universitário de Lisboa, 1649-026 Lisboa, Portugal; 2 Aptoide, S.A., 1600-196 Lisboa, Portugal; 3 CISUC—Center for Informatics and Systems of the University of Coimbra, 3004-531 Coimbra, Portugal

**Keywords:** machine learning, malware detection, mobile security

## Abstract

The widespread usage of mobile devices and their seamless adaptation to each user’s needs through useful applications (apps) makes them a prime target for malware developers. Malware is software built to harm the user, e.g., to access sensitive user data, such as banking details, or to hold data hostage and block user access. These apps are distributed in marketplaces that host millions and therefore have their forms of automated malware detection in place to deter malware developers and keep their app store (and reputation) trustworthy. Nevertheless, a non-negligible number of apps can bypass these detectors and remain available in the marketplace for any user to download and install on their device. Current malware detection strategies rely on using static or dynamic app extracted features (or a combination of both) to scale the detection and cover the growing number of apps submitted to the marketplace. In this paper, the main focus is on the apps that bypass the malware detectors and stay in the marketplace long enough to receive user feedback. This paper uses real-world data provided by an app store. The quantitative ratings and potential alert flags assigned to the apps by the users were used as features to train machine learning classifiers that successfully classify malware that evaded previous detection attempts. These results present reasonable accuracy and thus work to help to maintain a user-safe environment.

## 1. Introduction

Smartphones, tablets, and other mobile platforms have long been integrated into our daily lives due to the combination of portability, high computational power, Internet access and usability, making them necessary tools in our society. These personal computing devices, surpassing 14 billion units sold in 2020, have stimulated the development of sophisticated mobile malware. McAfee detected more than 35 million malware samples in 2019 [1].

With Android being the most used mobile operating system (OS), with roughly 76% of the global market share as of November 2019 [2], due to its open-source approach and a free-of-charge integrated development environment (IDE) that provides developers an easier access to its platform. On the other hand, iOS, which has rigorous approval policies and requires developers to use proprietary hardware and software to develop and publish iOS apps, places a higher entry barrier to those wishing to develop for the iOS ecosystem. In addition, the Android platform also allows its users to install apps from unverified sources that may be available on the Internet and from third-party app stores.

The Android platform’s ease of use and developer friendliness, together with the fact that these apps manage large volumes of sensitive and personal information (e.g., financial or messaging apps), makes the Android mobile ecosystem an ideal target for malware developers. In an informal view, malware is any software intentionally designed to cause damage to a device or client. In 2013, a report showed that attackers could earn up to USD 12,000 per month with mobile malware [3]. The rise of mobile malware can be, in part, linked to the development of new technologies providing new access points and attack vectors for profitable exploitations [4]. In addition, an increase in black markets that sell system vulnerabilities, malware source-code and malware development tools have provided a more significant incentive for profit-driven malware [5].

The most predominant Android app stores have developed malware detection methods in order to filter through the published apps and block those deemed malicious to protect its users. They achieved this by using either static or dynamic malware detection methods, or a conjunction of both, to scan the app’s intent and behavior to determine if it should be classified as malware or not. Unfortunately, all these methods have a failure rate and can be bypassed. For example, static analysis detectors that rely on the simple analysis of the application code are vulnerable to code obfuscation techniques that remove or limit code access, such as string encryption or renaming methods and variables [6]. Dynamic analysis, which focuses on observing the application behavior during runtime, remains vulnerable to the use of native code (e.g., non-Java code compiled to run on an Android CPU) or reflection (e.g., modifying methods, classes and interfaces during runtime) [7].

In some cases, certain malicious applications can detect emulated environments and suppress their malicious behavior accordingly in order to avoid detection, as found by Xu et al. [7]. Even with a combination of both methods, hybrid malware analysis still fails to address these issues completely. Furthermore, as malware constantly evolves to find new exploits and attack vectors, many malicious applications will bypass the detection strategies mobile market operators put in place. Due to the growing risk of malware developers bypassing safeguarding mechanisms, further and different mechanisms must be added to the already existing malware detection and mitigation methods. An in-depth look into the provided applications’ user feedback for apps that are later flagged as malware can reveal trends that help identify these malicious applications that otherwise are able to evade detection.

In this paper, we aim to study the effectiveness of user applications’ feedback when turned into features to train machine learning algorithms for malware detection in the Android environment. To devise the best approach for features and algorithm selection, we apply several data analysis methods, data preprocessing techniques, and machine learning approaches to train and evaluate the performance of different malware classifiers for Android apps. This model became part of a broader system that already includes several different components of pre-release verifications. Still, there is a minor number of applications that, even after passing the internal system, are withdrawn by the QA team, after being signaled by the users.

To accomplish this study, we used real-world user data provided by an app store, Aptoide (https://en.aptoide.com), to mimic user traffic in a large-scale app distributor. Aptoide is one of the largest Android app stores, acting as an alternative to Google Playstore, with over 250 million unique active users and partnerships, 15,000 app developers and over 1 million apps available for users to download and install on their devices. This research was proposed by Aptoide in order to improve their malware detection system and raise its safety levels.

Even though malware is easily defined as software that can harm users, it is a difficult concept to isolate in a tight definition. Some malware variants are clearly labeled, such as those that steal the user’s private information. However, software exhibiting numerous of advertisements can also be classified as malware. In this view, this paper considers as ground-truth for the *malware* concept the software annotated as such by the Aptoide professional experts. A more detailed view of this dataset can be found in [8].

The remainder of this paper is structured as follows. The related work is described in Section 2. Section 3 describes the data used and how their features relate. Next, the methodology for building the machine learning model is described in Section 4, which pinpoints the various forms of data preprocessing and machine learning algorithms used. Next, the experimental setup section (Section 4.3) presents the experiments, as well as their results and comparisons. Finally, the last section presents the conclusions and future work.

## 2. Related Work

Research on malware detection methods for Android is built on decades of traditional signature-based, static and dynamic malware analysis research developed for personal computers (PCs). Although traditional techniques such as virtualization and decompiling are also used in mobile environments, the specifics of virtual machine (VM) architectures and code packaging differ from their PC counterparts. So, traditional methods need to be enhanced to deal with similar threats.

The majority of malware detection methods are based on the traditional signature comparison. Using an extensive database of known malware signatures enables a program to detect the presence of malware by matching its byte code patterns with those available in the database. Unfortunately, this implies that users are not safe from malware that has not yet been detected and added to these databases, or from malware that has slightly changed their code signature. Therefore, the rapid growth of malware apps indicates that this approach, although useful, tends to lag.

Other traditional methods, such as static and dynamic code analysis that examine the app’s source-code and the products of its execution, respectively, also have their limitations and drawbacks, as reported by Tam et al. [9]. The major drawback of these approaches is the obfuscation techniques that remove or limit source-code access [6]. Other drawbacks include the injection of non-Java code, network activity, and the modification of objects at runtime [9].

Recently, some studies have pointed to the possibility of identifying malware based on user feedback, but the focus is mainly on user textual user reviews. Hadad et al. [10] used data similar to those used in this study (2500+ application reviews from a well-known app store) and text mining techniques to analyze the review’s texts and create a training set based on these characteristics. On the other hand, ref. [11] performed text summarization and sentiment analysis using 17 applications’ reviews and, based on interviews, concluded that mining the review texts can indeed help to understand the security issues found in applications. The usage of user applications’ feedback may prove helpful regarding malware detection, although very little work has been done in this direction. For example, WHYPER [12] focused on processing app market metadata, such as application descriptions, to examine whether the description provided any indication as to why the application needed specific permissions. In addition, it also helped to conduct risk assessment of mobile applications using natural language processing (NLP) techniques. Other works that attempt some kind of mining of user feedback employ it for raising generic functional or maintenance requests [13,14,15,16]. In a very recent survey, Ebrahimi et al. [17] also referred that, so far, only a few works attempted to mine user privacy concerns from app store reviews.

Nonetheless, parallels can be drawn from other domains that have also felt the need to analyze users’ feedback, such as hotels [18], restaurants [19], and e-commerce providers [19]. With app store users being able to post their feedback regarding their downloaded apps, via ratings, comments, and other sorts of feedback, this can potentially be used to help enhance the performance of machine learning classification methods by providing more features to be considered in the analysis.

## 3. Data

**Figure 1:**
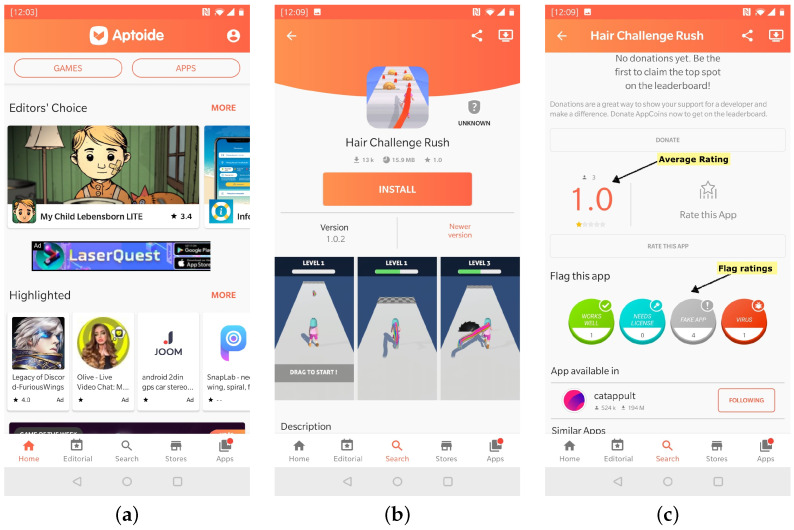
Aptoide application, with app user reviews and the app flags classification. (**a**) Aptoide’s app landing page. (**b**) Chosen app view. (**c**) App’s rating and flags.

Analyzing the relationship between user feedback information/rating and malware can help identify patterns that indicate the presence of malware in a given application. This section details the main associations between malware and user feedback that enable the detection of malware patterns. The analysis is based on three months of user feedback historical data provided by Aptoide and is composed of users’ ratings and flags, as well as the QA team response. This dataset refers to 2332 applications from all kinds of mobile application genres released between 1 August to 31 October 2019. The feedback data were collected from the beginning of October 2019 till the end of January 2020. For each one of the apps, the star ratings range between one (1), the lowest rating, and five (5), the highest. The features used are as follows: the number of star ratings of each type it obtained from the users; the quantity of “Good” flags; the quantity of “Virus” flags; the quantity of “Fake” flags; and the quantity of “Needs License” flags (Figure 1c). In addition, the Aptoide security professionals also labeled each one of the involved apps as “trusted” or “critical”, where “critical” means it was identified as malware.

The statistical distribution of the apps’ rating feedback is given in Table 1. Each sample contains the counts of feedback from users for a given app. The minimum number for each one of the features is always zero, and all apps have at least one rating of each classification from 0 to 5. The mean column in Table 1 shows the average number of the ratings or flags observed for all the apps. The maximum is the total count of a given rating or flag observed on an app that exceeds the number of votes for that rating on any other app. The features’ distribution reveals a high degree of deviation from the mean and variation of ratings/flags given by app users. This can be explained by the fact that only a small percentage of the apps reach high levels of popularity, thereby receiving more feedback, whether favorable or not. In fact, one can observe from these figures that most users are more inclined to give feedback based on strong opinions, and that positive strong opinions predominate.

Assuming that the feedback is evenly distributed among all application types, a correlation between all the features can be analyzed in advance. The relationship between variables becomes evident by joining all the training data samples. Table 2 shows the correlation matrix of all features (ratings and flags) using the relationship between the correlation coefficient matrix and covariance matrix. Table 2 shows that ratings express a high degree of correlation between them, which is also valid to a lesser extent for flags. However, correlation values between ratings and flags are relatively low. The correlation between the lowest rating values, Rating 1 and Rating 2, is the highest, but since these have different correlation values with the other variables, it was decided not to merge them to make the most use of the limited data available. Since all these features show different forms of correlation, they should be used to avoid information loss.

### Dimensionality Reduction

**Figure 2:**
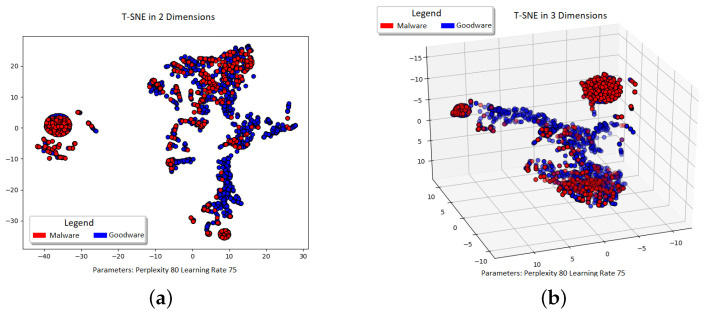
t-SNE projection views. (**a**) t-SNE 2D projection group formation. (**b**) t-SNE 3D projection view with cluster separation.

Another way to look at the data is by using dimensionality reduction techniques to enhance visualization. In order to gain early insight into the data structure, T-distributed stochastic neighbor embedding (t-SNE) was used. This is a nonlinear dimensionality reduction technique that allows to check if the data are separable before the application of classifiers. Several algorithm runs were tested with different parameter combinations, namely, perplexity, number of components, and learning rate, to verify the parameter impact and stability in results. Figure 2a,b shows one of the algorithm’s outputs. The chosen examples are good representatives of most parameter combinations, using either two (Figure 2a) or three components Figure 2b), respectively. Whilst Figure 2 shows the spread clusters of mixed data, several concentrated clusters of malware-labeled samples can be observed. This preliminary analysis shows that, using only user feedback, there are indeed identifiable malware trends, even if using only three or fewer components.

## 4. Model Implementation

In this section, the implementation of the machine learning models is discussed. All of these experiments were performed using Python’s scikit-learn package and XGBoost. Several machine learning classifiers were used for comparison: XGBoost (XGB); random forest (RF); support vector machines (SVM); K-nearest neighbors (KNN). None of these algorithms expresses prior assumptions about the data.

### 4.1. Data Processing

Before applying any of the previously mentioned methods, the dataset was prepared to avoid model overfitting and biases to obtain more accurate results when applied to similar data. With the assumption that malware applications do not last long enough in the marketplace before being detected and that, between the trusted applications, a small percentage of top-rated apps that were available for a long time in the market and accumulated a lot of user feedback in comparison to the majority of trusted applications, it was decided to drop the top 1% of the data population for each of the variables (that is, 56 apps were removed). This change reduced the dataset to 2276 app samples. The statistical distribution after removal can be seen in Table 3. It can be observed that, despite the decrease in the number of samples, the displayed statistics in Table 1 follow the ones depicted in Table 3 although now presenting much lower standard deviations. Although the dataset now comprises 2276 samples, only 584 of these were labeled as malware and the remaining 1692 were labeled as trusted. A random undersampling was applied to trusted samples to deal with such imbalance. This allowed a ratio of 1:1 between malware and trusted applications, providing a final training dataset of 1168 samples. Although the sample size was effectively halved, this helps to avoid a trusted application bias.

Several feature preprocessing methods were selected in sequence for use with each classifier to select the one that yielded better results on a case-by-case basis. Preprocessing assures the best performance possible on distance-based algorithms, such as KNN and SVM, and reduces the training time on those not impacted by sample distances. Classifiers were also trained on the dataset without any preprocessing for comparison. The preprocessing techniques used were as follows: no preprocess method for baseline comparison (NoPrep); standard scaler (STD), which standardizes features by deducing the mean and scaling to unit variance; normalizer (NORM), which normalizes features individually to the unit norm, converting values to a 0 to 1 scale; Yeo–Johnson power transformer (PowerYJ) [20], which applies a feature-wise power transform to make the data more Gaussian-like; and a quantile transformer (Quant), that transforms features using a cumulative distribution function to map the original values to a uniform distribution.

### 4.2. Classification Algorithms

After the data were processed, several classification algorithms were used to predict a binary classification, discriminating between malware or trusted applications. The algorithms used were support vector machine (SVM) that uses a kernel function to handle nonlinearly separable data. In this case, due to the data distribution previously seen (Figure 2), a radial basis function (RBF) kernel was used. The k-nearest neighbour algorithm (KNN) checks the k-nearest sampled features using Euclidean distances. It classifies an instance according to a plurality vote of its neighbors. Thirdly, the random forest (RF) classifier, which belongs to the family of ensemble classifiers, operates by building a large number of decision trees and returns the class corresponding to the mode of the classes of each of the individual trees. Finally, the extreme gradient boosting algorithm (XGBoost), again an ensemble method, is also an optimized gradient boosting framework.

### 4.3. Experiments and Results

#### Experimental Setup

This section explains the process by which the optimized classifier results were obtained. In order to optimize each of the previously mentioned classifiers, a framework was developed to avoid the common pitfalls of overfitting and feature biases. Firstly, the data were divided into two sets, 20% to approximate the actual error and the remaining 80% to train and validate employing the stratified K-fold cross validation technique and setting k=10. The stratification is achieved by preserving the relative percentage of samples for each one of the classes. Doing so ensures that each training set fold distribution remains as close as possible to the one in the entire working dataset. This setting was run using a random grid search to test 500 parameter combinations for each classifier. The model using the more effective combination of parameters is returned under a reasonable timescale without testing every possible combination in a regular grid search.

This framework was applied together with each preprocessing method mentioned earlier, alongside with the application of the framework using a dataset without any preprocessing for comparative analysis.

### 4.4. Results

In order to evaluate model performance, standard metrics were used for analysis and comparison. Namely, the $F_{1}$ score or the harmonic average of precision and recall, *accuracy*, *false positive rate*, *false negative rate*, *receiver operating characteristic (ROC)/area under curve (AUC)* score, as well as *precision-recall (P-R)* AUC. We used a weighted macro-average of these individual metrics to measure each model’s overall classification performance and adjust for classification biases. The total time taken for each model’s random grid search was also considered to estimate the model’s training time efficiency.

Table 4 presents the results for all classification algorithm models over differently preprocessed datasets. All algorithms present similar performance-wise results with minor variations. SVM and KNN show better results with the PowerYJ preprocessed dataset, with $0.75F_{1}$ score and 0.818ROC/AUC and 0.83ROC/AUC, for SVM and KNN, respectively. Note that the KNN models’ training runtimes are relatively lower when compared with the SVM’s training times, making it the most effective model time-wise, even though the evaluation of KNN is bound to be slower than SVM. Comparatively, although presenting higher times for the training, ensemble algorithms XGBoost and RF achieved the best results overall, with XGBoost coming out on top, both for accuracy, F1-measure and AUC. Moreover, the training time is quite competitive for the higher results (using PowerYJ).

Notoriously, the PowerYJ preprocessed dataset, in general, shows a higher performance when compared with the remaining preprocessing methods, and, as expected, when compared to the no-preprocessing run. Table 5 highlights the difference between the best methods, highlighting the preprocessing that enabled the models to achieve the top scores. Table 5 also presents the mean of the cross-validation results and their standard deviation (SD). The low standard deviation values for all models indicate a small variance between each fold from the cross-validation procedure, suggesting that the algorithms could be generalized for similar datasets.

Results indicate that (a) the classification analysis of users’ feedback is feasible and that (b) near 72% of the malicious apps can be detected by using only given ratings and potential flags. Thus, the results prove that using user’s feedback ratings and flags can assist in identifying malware after it succeeds in bypassing the more traditional static and dynamic malware analysis.

## 5. Conclusions and Future Work

A still significant number of apps is still able to bypass the classic (static and dynamic) malware detection techniques and become available in the marketplace for any user to download and install. This paper studies the usefulness of user feedback on apps already available in an actual Android marketplace to predict Android malware, using machine learning classifiers. By using a robust methodology with different preprocessing techniques and a random grid search parameter function, this paper establishes the expected classification rates for the detection of malicious applications using algorithms trained solely in user quantitative feedback patterns present in the users’ feedback data, that is, without inspecting each of the applications’ code or runtime behavior. The best model was trained with XGBoost and was able to surpassed other models in most of the metrics used to evaluate performance for this task, even without any data preprocessing. Still, random forest and distance-based algorithms such as KNN and SVM show interesting results in the best scenarios.

Several avenues can be explored for future research. Firstly, we can study the best way to integrate this tier of classification with pre-deployment, static and dynamic analysis. Secondly, the application of natural language processing techniques to extract other features from the comments users leave on the app store that may be relevant for training alongside flags and ratings for each app is likely to increase precision and should be studied. Thirdly, a broader timescale of user feedback data would enable an analysis of the evolution of the models’ results to look for possible trends and shifts in app stores. More data availability will allow for a comparison with different experimental setup parameters and different ratios between malware/non malware apps that better mimic real-world scenarios. Finally, a study of model performance decay and automated model replacement is underway and will certainly complement these results and allow for a fully automated system.

## Figures and Tables

**Table 1:** Statistics regarding ratings and flags of the provided data set.

	Mean	Standard-Deviation	Maximum
Rating 1	543.22	4071.58	37,049
Rating 2	146.46	1087.31	9859
Rating 3	1085.56	8213.47	74,968
Rating 4	555.01	3418.38	29,994
Rating 5	5100.14	38,542.78	353,411
Flag Good	6.72	41.52	1021
Flag Virus	3.48	14.33	244
Flag Fake	3.62	18.67	367
Flag License	9.13	110.99	2417

**Table 2:** Matrix showing the Pearson correlation between variables.

		Ratings					Flags			
		Rating 1	Rating 2	Rating 3	Rating 4	Rating 5	Flag Good	Flag Virus	Flag Fake	Flag License
Ratings	Rating 1	1.00	0.95	0.85	0.81	0.882	0.17	0.09	0.02	0.11
	Rating 2	0.95	1.00	0.86	0.91	0.895	0.13	0.06	−0.01	0.06
	Rating 3	0.85	0.86	1.00	0.66	0.961	0.170	0.068	0.000	0.073
	Rating 4	0.81	0.91	0.66	1.00	0.755	0.06	0.04	−0.02	0.05
	Rating 5	0.88	0.90	0.96	0.76	1.000	0.17	0.07	−0.01	0.06
Flags	Flag Good	0.165	0.13	0.17	0.06	0.17	1.00	0.62	0.59	0.54
	Flag Virus	0.09	0.06	0.07	0.04	0.07	0.62	1.00	0.65	0.41
	Flag Fake	0.02	−0.01	0.00	−0.02	−0.01	0.59	0.65	1.00	0.40
	Flag License	0.11	0.06	0.07	0.051	0.06	0.53	0.41	0.40	1.00

**Table 3:** Distribution of ratings and flags after dropping the top 1% apps. Each sample represents.

	Mean	Standard-Deviation	Maximum
Rating 1	72.34	246.73	2461
Rating 2	21.79	82.69	697
Rating 3	145.91	553.07	5761
Rating 4	176.76	950.82	11,375
Rating 5	718.54	2695.08	27,967
Flag Good	3.46	8.46	106
Flag Virus	2.29	4.16	38
Flag Fake	2.10	5.44	74
Flag License	0.98	3.59	48

**Table 4:** Comparison of model performance.

Model	Preprocess Method	*F*_1_	Accuracy	FPR	FNR	ROC AUC	P-R AUC	Total Time Taken
XGBoost	NoPrep	0.770	0.775	0.136	0.314	0.867	0.835	1 h 40 m 15 s
	STD	0.770	0.775	0.144	0.305	0.873	0.841	1 h 27 m 51 s
	NORM	0.750	0.754	0.203	0.288	0.842	0.775	0 h 56 m 20 s
	PowerYJ	0.790	0.788	0.144	0.28	0.863	0.808	0 h 36 m 36 s
	Quant	0.770	0.771	0.136	0.322	0.869	0.839	0 h 23 m 17 s
RF	NoPrep	0.720	0.725	0.237	0.314	0.808	0.789	1 h 8 m 46 s
	STD	0.710	0.712	0.212	0.364	0.804	0.789	3 h 49 m 1 s
	NORM	0.730	0.733	0.314	0.22	0.81	0.771	3 h 26 m 28 s
	PowerYJ	0.710	0.712	0.229	0.347	0.806	0.79	3 h 11 m 43 s
	Quant	0.720	0.72	0.237	0.322	0.806	0.789	3 h 5 m 55 s
KNN	NoPrep	0.710	0.716	0.212	0.356	0.774	0.767	0 h 1 m 1 s
	STD	0.700	0.699	0.254	0.347	0.771	0.744	0 h 1 m 9 s
	NORM	0.700	0.703	0.246	0.347	0.796	0.777	0 h 1 m 11 s
	PowerYJ	0.750	0.754	0.212	0.28	0.83	0.807	0 h 1 m 1 s
	Quant	0.720	0.725	0.237	0.314	0.796	0.647	0 h 1 m 3 s
SVM	NoPrep	0.720	0.716	0.314	0.254	0.787	0.743	1 h 13 m 59 s
	STD	0.600	0.614	0.542	0.229	0.721	0.716	0 h 33 m 23 s
	NORM	0.700	0.699	0.246	0.356	0.79	0.771	0 h 25 m 56 s
	PowerYJ	0.750	0.75	0.186	0.314	0.818	0.794	0 h 29 m 25 s
	Quant	0.710	0.712	0.186	0.39	0.769	0.714	0 h 32 m 6 s

**Table 5:** Comparison of top model performance for each algorithm (SD—standard deviation).

Model	Preprocess Method	Statistic	*F*_1_	Accuracy	FPR	FNR	ROC AUC	P-R AUC
XGBoost	PowerYJ	Mean	0.790	0.788	0.144	0.28	0.863	0.808
		SD	0.045	0.029	0.022	0.032	0.036	0.27
RF	NORM	Mean	0.730	0.733	0.314	0.22	0.81	0.771
		SD	0.040	0.038	0.048	0.33	0.048	0.042
KNN	PowerYJ	Mean	0.750	0.754	0.212	0.28	0.83	0.807
		SD	0.033	0.031	0.046	0.037	0.055	0.049
SVM	PowerYJ	Mean	0.750	0.75	0.186	0.314	0.818	0.794
		SD	0.049	0.023	0.022	0.051	0.053	0.048

## Data Availability

Data used in this study were graciously provided by Aptoide S.A. within the scope of project AppSentinel.
